# “If children don't feel safe, they won't come back”: A qualitative exploration of parents' perceptions of health coordinators in a family-based programme in socially disadvantaged communities

**DOI:** 10.1016/j.puhip.2024.100575

**Published:** 2024-12-21

**Authors:** Lisette Farias, Mai-Lis Hellenius, Gisela Nyberg, Susanne Andermo

**Affiliations:** a Karolinska Institutet, Department of Neurobiology, Care Sciences and Society, Division of Nursing, 141 83, Huddinge, Sweden; b Karolinska Institutet, Department of Neurobiology, Care Sciences and Society, Division of Occupational Therapy, 141 83, Huddinge, Sweden; c Karolinska Institutet, Department of Medicine, 171 77, Solna, Sweden; d The Swedish School of Sport and Health Sciences, Department of Physical Activity and Health, Lidingövägen 1, 114 33, Stockholm, Sweden; e Karolinska Institutet, Department of Global Public Health, 171 77, Stockholm, Sweden

**Keywords:** Access to physical activity, Health disparities, Health promoters, Socioeconomic status

## Abstract

**Background:**

Families residing in disadvantaged communities encounter inequalities that restrict their engagement in physical activity. Family-based interventions and health coordinators have been proposed as promising approaches to encourage physical activity among parents and children. However, there is a paucity of knowledge regarding family experiences of such programmes and the ways health coordinators facilitate continued participation in programmes delivered in disadvantaged communities. The study aimed to explore parents’ perceptions of health coordinators in a family-based physical activity programme, Open Activities, delivered in disadvantaged communities in Sweden.

**Study design:**

An exploratory design with a qualitative ethnographic approach.

**Methods:**

Multiple methods, including 12 interviews, 15 observations and field notes, and prolonged researcher engagement between February 2022 and December 2023, were used to obtain complementary insights into parents’ perceptions and experiences in the Open Activities programme. Data was analysed using reflexive thematic analysis.

**Results:**

A main theme and three sub-themes emerged from the analysis. The sub-themes reflect participants’ perceptions of how health coordinators provide a sense of safety for parents and children, making them feel appreciated and motivated to continue participating in the programme. The sub-themes also reflect the struggles that families encounter with security and social disorders in their neighbourhoods and how these difficulties influence their participation in outdoor activities and trust in outside people, including health coordinators. This required health coordinators to demonstrate a deep commitment to their communities, cultivate trust and fairness, and take a more assertive role in enforcing rules and ensuring respect.

**Conclusion:**

The involvement of health coordinators, aware of the issues facing disadvantaged communities, could represent a promising avenue for advancing health equity through physical activity. Failure to consider the potential of health coordinators to promote safety can compromise programmes’ sustainability and even exacerbate existing disparities.

## 1 What this study adds

• In addition to previously described economic, religious, and cultural barriers, safety concerns and a general lack of trust in outsiders represent significant obstacles to physical activity in disadvantaged communities.
• Health coordinators need to establish a sense of order, fairness, and trust to involve and retain difficult-to-reach groups in family-based programmes.
• For health coordinators, a strong commitment facilitates the promotion of physical activity and equity in disadvantaged communities.

## 2 Implications for policy and practice

• Promoting outdoor safe spaces can increase the participation of disadvantaged groups in physical activity.
• Using health coordinators to promote physical activity among difficult-to-reach groups can counteract existing disparities.

## 3 Introduction

Despite the multiple benefits of physical activity (PA) for the physical and psychosocial health of adults and children [1], few reach the recommended levels of PA worldwide [2]. International evidence shows that in disadvantaged neighbourhoods, families with low socioeconomic status (SES) face significant inequalities that limit their access to PA [3]. These barriers include a lack of safety and safe outdoor spaces [4,5] and sports facilities [6]. Consistent with this evidence, children from low SES families in Sweden are less likely to participate in organised sports and play outdoors than children from high SES families [7]. Barriers to families' participation in PA include parents’ economic constraints, lack of time due to multiple shifts/jobs [8] and low priority of PA [9]. This means that particularly children from low SES families do not have the same possibilities to start a healthy life due to the social and health disparities existing in their disadvantaged areas. Thus, improving support for underserved groups could be a powerful strategy to establish the roots of healthy behaviours and social support in childhood development through engagement in PA [6].

Environmental contexts, such as family, school, and local communities, can influence children's engagement in PA [10]. Parents, in particular, can exert a significant influence by modelling their children's lifestyles, eating habits and PA [11,12] when provided with the necessary support [13]. To support positive, healthy lifestyle changes and engagement in 10.13039/100006131PA, the utilisation of health coordinators has been proposed [14]. Health coordinators are commonly referred to as lay health educators, community health advisors, or health workers [15]. They play an essential role in recruiting, retaining, and providing access to participants living in marginalised difficult-to-reach, and underserved or disadvantaged communities [15]. Studies have reported that working as a health coordinator can increase coordinators' self-esteem, sense of duty [16], community prestige, and satisfaction in helping others [15]. While the significance of health coordinators is evident, there are still knowledge gaps in the perception of health coordinators promoting PA [14] and working with families in disadvantaged or hard-to-reach communities [17]. Access to PA is essential for these communities because they face significant health inequalities, such as unequal access to health-promoting activities and nutritious foods [18]. This contributes to their higher rates of chronic disease, lower life expectancy [19] and high burden of disease and disability [20]. In addition, these communities have high rates of unemployment [20], which contributes to perceived stigma and marginalisation [21].

To develop family-based PA programs that address the social and health inequalities that affect disadvantaged communities, it is critical to explore parents’ perceptions of the role of health coordinators in promoting PA. This, in turn, can increase adult and child participation in PA, promote positive, healthy lifestyles, and reduce health disparities in these areas.

This study explored parents’ perceptions of health coordinators in a family-based PA programme, Open Activities, delivered in disadvantaged communities in Sweden.

## 4 Methods

### 4.1 Study design

The study used an exploratory design with a qualitative ethnographic approach [22] to explore parents' perceptions of health coordinators who lead the family-based Open Activities program. The use of multiple methods, including observations [23], field notes, interviews, and prolonged researcher engagement, is consistent with the study's social constructivist philosophy. This combination or triangulation of methods supported a deeper understanding of participants' interactions with health coordinators and the social construction of their dynamics within a specific cultural context [22].

### 4.2 Study setting

The study involved observations, field notes, and extended research engagement in a suburban disadvantaged community in Stockholm, Sweden. It also included interviews with parents from disadvantaged communities in Sweden, where the Open Activities programme was implemented.

The programme, which started in September 2021, offers free drop-in outdoor activities for families with children living in selected disadvantaged areas in 16 municipalities in Sweden. Families are invited through flyers posted around the community and by the health coordinator, who goes around the area 30 min before the activities start. All families are welcome to join the activities with their children and siblings. A health coordinator leads the activities, which take place every weekend for 1 h. The programme includes 45 min of activities, and the remaining 15 min are dedicated to answering quizzes about healthy habits and offering fruit to families. The activities varied each weekend and included games such as football and team games such as Red Rover, Capture the Flag or different versions of Tag. Parents and children are mixed in the teams. The average number of participants per weekend was approximately 25–35.

### 4.3 Study participants and recruitment

Purposive sampling was employed to recruit a total of 12 parents (at least or older than 18 years) who had participated with their families (at least one child) in the Open Activities programme in 2022–2023 and were willing to share their perceptions of the health coordinators leading the activities. The participants (9 female and 3 male) lived in socially disadvantaged areas characterised by the prevalence of physical inactivity, sedentary behaviours, smoking, and a high population of people of non-native backgrounds [24].

The researchers presented the study in writing and orally to potential participants who contacted them directly or through the health coordinators to schedule an interview at a location and time that suited them. Six participants preferred to be interviewed immediately after the activities (one of these participants required the assistance of a friend due to language barriers) in a public park facility to ensure privacy. One requested that questions be answered via email, and five participants asked to be interviewed over the phone due to time constraints.

### 4.4 Data collection

Data was collected between February 2022 and December 2023. Observations (n = 15) and prolonged involvement of researchers in the programme were conducted during February and May 2022. Researchers participated in the programme sessions for 1–1.5 h on 15 Saturdays to familiarise themselves with the activities provided, the dynamics among participants, and the interactions between participants and health coordinators. Researchers’ level of involvement was moderate to active [23], meaning they interacted with participants without interrupting the activities. Field notes were taken after participation in the activities using a grid elaborated for this study and based on ethnographic principles to document the sessions [22].

The researchers conducted interviews using a semi-structured guide developed for this study, guided by the aim and their previous programme evaluation experiences. The guide contained ten open-ended questions, allowing participants to share their experiences comfortably. The topics included in the semi-structured interviews were related to a) previous experiences of PA outdoors, b) experiences participating in the Open Activities programme, c) the potential impact of the programme on their personal and family participation in PA, and d) facilitating and hindering factors for participating in the programme and outdoor PA. Examples of questions are: how would you describe the Open Activities programme? Can you describe a situation or session that was experienced as engaging or fun and one that was experienced as less good or engaging during the programme? What has it meant for you or your family to participate? Eleven participants preferred conducting interviews in Swedish, while only one preferred English. The interviews lasted an average of 25–46 min and were audio-recorded and transcribed by the researchers.

### 4.5 Data analysis

The collected data was analysed using Braun and Clarke's reflexive thematic analysis (TA) six-phase process [25,26]. These six-phases guided the analysis, providing flexibility and constant movement back and forth through the phases. The interviews were transcribed and re-read several times to familiarise oneself with the data. The field notes and observations were used to obtain a better understanding of the activities provided by the programme and the interactions among participants and health coordinators. Yet, these were not included in the coding and development of themes. During the coding process, notes were taken to make sense of the data as a whole, supporting researchers' reflexivity. The Atlas. ti 23 program was used to generate notes and initial codes and to organise the data into preliminary themes. At this stage, the authors discussed these themes until a consensus was reached based on the data and their previous involvement with the programme as evaluators. Then, these preliminary themes were reorganised based on shared meanings. They were then checked against the entire dataset to produce a thematic map of the analysis. The authors discussed this mapping until they reached a consensus on the final themes.

### 4.6 Ethical consideration

Ethical clearance was obtained from the Regional Ethical Review Board in Stockholm (Dr no: (2022-02643-02). The participants were recruited in the communities where the programme was implemented. The researchers provided verbal and written information about the study and their right to withdraw at any time during the study period. They also agreed with the participants on a suitable time, date, and venue for an interview. Before data collection, informed consent and permission to use an audio recorder were obtained from participants.

## 5 Results

**Fig. 1:**
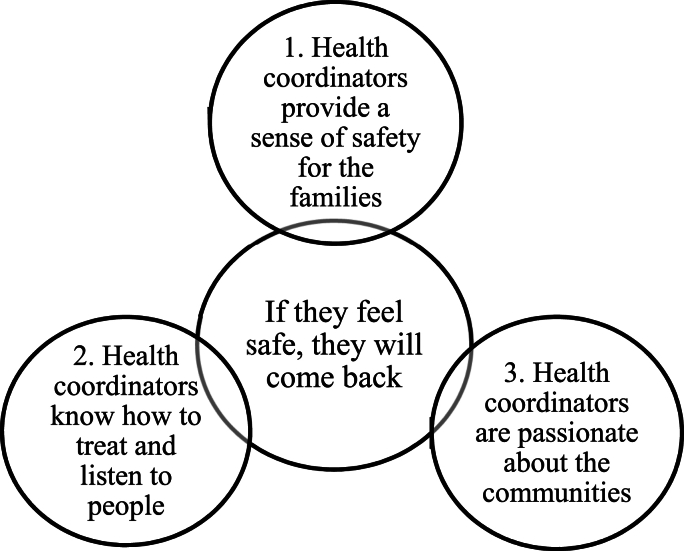
Main theme and three sub-themes.

The reflexive thematic analysis revealed a main theme and three sub-themes (Fig. 1). The main theme, ‘If they feel safe, they will come back,’ reflects parent's perceptions of health coordinators as pivotal in providing their children a sense of safety and trust. This safety and trust were vital for parents, the primary gatekeepers to children, to continue participating in the programme. The sub-themes were ‘Health coordinators provide a sense of safety for the families’, ‘Health coordinators know how to treat and listen to people’, and ‘Health coordinators are passionate about the communities.’ The participants' quotes were translated, keeping grammar errors to reflect the participants' language level.

The characteristics of the participants are presented in Table 1.

**Table 1:** Participants characteristics

Participant	Age	Number of children	Civil Status	Profession/work status
1. **Female**	43	4	Married	Preschool teacher assistant
2. **Female**	37	4	Married	Studying Swedish as a second language
3. **Male**	44	3	Married	Research Specialist
4. **Female**	48	3	Married	Preschool teacher assistant
5. **Female**	33	2	Married	Parental leave, preschool teacher assistant
6. **Female**	48	3	Married	Preschool teacher assistant
7. **Male**	43	2	Married	Export operations
8. **Female**	33	3	Married	Part-time worker in-home care and student
9. **Female**	39	3	Married	Part-time worker as a doula and student
10. **Female**	49	3	Married	Studying full-time
11. **Female**	48	4	Married	Assistant nurse
12. **Male**	48	3	Divorced	Warehouse worker

### 5.1 Main theme: if they feel safe, they will come back

#### 5.1.1 Sub-theme 1: health coordinators provide a sense of safety for the families

Parents mentioned that their perception of their neighbourhood as unsafe has increased due to various incidents in the area (e.g., crime, violent protests, gangs). For this reason, parents stressed the importance of ensuring that their children feel safe during the activities, given that they take place outdoors.

*The most important thing in these areas is the security when they [children] feel that, they come to play at the programme if they feel safe then these children will stick to this programme if they don’t feel safe then they won’t come back* (Interviewee 5)

Parents emphasised that previous events had increased their insecurity and made them less open to trusting people outside their community, including health coordinators. One parent stated, “*If I don't feel safe. I shouldn't go”* (interviewee 11). For some parents, trusting the health coordinator was critical to continued participation in the activities. In particular, some participants described male coordinators as untrustworthy due to social stereotypes, cultural norms, and past events in their community.

*They [parents] are careful about having a male health coordinator after what is happening here in the neighbourhood. When it’s only male, you don’t trust even though this person may be the best in the world* (interviewee 1)

#### 5.1.2 Sub-theme 2: health coordinators know how to treat and listen to people

To promote safety and trust, the health coordinators sometimes took on a firmer role to ensure that rules and respect were followed within the group. One parent said, “*She is very nice and social, straight, but the children feel secure”* (interviewee 5). This ‘firmer role’ was particularly important given the social challenges, such as protests and vandalism, occurring in their neighbourhood. According to parents, health coordinators *“know how to treat someone*” (interviewee 11), as they can be both gentle and firm when necessary.

*Our health coordinator has great control everywhere when someone talks, she listens actively and doesn’t allow anyone else to interrupt, and we have to raise our hands to be able to speak. It’s great that she has this control. She is a great leader, so there are never any problems* (Interviewee 9)

Another important aspect was the health coordinators’ role being perceived as fair and caring for the children. One parent described this fairness as follows:

*One thing that she [the health coordinator] is good, she is fair. If it happens that the kids are sad, she always knows, listens, goes and takes the child, and takes care of him/her. She doesn’t just care. She cares so much* (Interviewee 11)

The health coordinators caring for the children was described by parents as, e.g., “*having a nice voice when talking with the children*” (Interviewee 4), “*being very adaptable. They listen very well to the children”* (Interviewee 10), and “*being professional with the children. They listen, it's not just play activities; they talk to them too*” (Interviewee 6).

#### 5.1.3 Sub-theme 3: health coordinators are passionate about the communities

As noted by the parents, some health coordinators had personal connections to these areas, such as having grown up there, being able to speak the language of the residents or having previously worked in the area. The parents described this passion as a commitment to their communities, which is necessary to build trust and safety in the face of social challenges such as those experienced in their neighbourhoods. This passion or commitment is also essential when health coordinators work mostly alone in these areas, where there is little or no support and a lack of opportunities for affordable after-school sports or physical activities for children.

*I thought it was great that the health coordinator, in this case, has taken the initiative for this area and seems to get things going. He [the coordinator] is really passionate about it, so it was a lot of fun, and the children thought it was great fun and that, yes, a fun thing, and then it created a cohesion out there* (Interviewee 10)

Some parents stressed that the health coordinator's strong commitment to their communities was reciprocal, meaning they would follow their coordinator if they were to leave. One participant stated, “*We want to keep our coordinator, absolutely; if she goes, then we will also go”* (Interviewee 11).

## 6 Discussion

The present study aimed to explore parents' perceptions of health coordinators in a family-based PA programme, Open Activities, delivered in disadvantaged communities in Sweden. The findings of this study illustrate the critical role of health coordinators in promoting safety to engage hard-to-reach and disadvantaged communities in PA. Hard-to-reach groups are often socially disadvantaged and low SES groups that are under-represented in health research due to a ‘culture of mistrust’ [27] or fear of authority, language or literacy problems, and lack of education/awareness of health promotion [28]. In the present study, feeling unsafe was one of the most important contextual barriers to accessing PA programs due to social disorders, violence, and previous incidents involving outside people. Previous research on adapting health promotion interventions to groups living in disadvantaged neighbourhoods emphasises the importance of allowing time to build trust [29] and identifying and addressing cultural, religious, or contextual barriers to access and participation [30]. The findings of this study highlight the critical role of health coordinators in providing a sense of safety to the families joining the program by taking a firmer role, somehow counteracting social disorders and ensuring that rules, order, and fairness were in place.

Another critical attribute of the health coordinators, as identified by participants in this study, is the ability to ‘know how to treat people’. A possible explanation for participants' appreciation of health coordinators' fair and gentle treatment, which should be expected, may result from previous experiences of inappropriate generalisations and potentially unsuitable programmes [31]. Failing to consider disadvantaged communities' specific challenges, health coordinators' decisions may be based on generalisations, stereotypes, and assumptions about these communities rather than on families' input [27]. Further, perceptions of stigmatisation [32] and social exclusion [33] can partly explain participants' appreciation and importance of being treated with gentleness and fairness by health coordinators. This study's participants described how health coordinators interacted with families as more than just leading the programme activities; it involved taking time to listen and talk to children and parents about their needs and preferences.

Finally, although outdoor/park-based PA in low SES neighbourhoods may attract individuals, programmes alone are insufficient to generate long-term participation in PA in these areas [34]. Consequently, the presence of health coordinators with personal connections to these communities can foster a robust commitment to equity, which can facilitate the establishment of trust. This could indicate that health coordinators play a pivotal role in bridging the knowledge imparted by health promotion programmes and the tacit and cultural knowledge among participating families in disadvantaged communities [35]. Nevertheless, participants in this study additionally described that they were so emotionally invested in their health coordinator that they felt a sense of dependency, stating that they would ‘go if the health coordinator goes’. Thus, establishing a sense of security and mutual trust within communities may have a dual effect, influencing positive and negative responses to utilising a health coordinator amongst families. These effects should be carefully considered when planning health coordinators and programme changes. Another consideration relevant to programmes in disadvantaged areas is the risk of homogenising these groups based on their socioeconomic status and neglecting other aspects of inequality, such as sexual orientation, gender, ethnicity, and disability. To avoid this neglect, it is recommended that intersectionality theory [36], which considers the intersection of individuals' multiple inequalities within social systems, informs the development of health promotion programmes and the training of health coordinators.

### 6.1 Limitations

The findings represent the perceptions of a relatively small number of participants in the Open Activities programme who were living in Sweden's suburban and socially disadvantaged areas. Therefore, these need to be carefully examined and do not represent parents' perceptions in general. Parents' positive perceptions about the health coordinators could be related to the fact that participants who were dissatisfied with the programme or health coordinators may have left the programme before researchers started to engage in the activities. This may explain why alternative or more contrasting perceptions are missing in participants' descriptions. Another potential explanation is that parents wanted to provide positive feedback regarding health coordinators so that they would not ‘lose’ their continuity in the programme due to the lack of programmes and extra activities for children in these areas.

Further, the fact that all researchers are female could have hindered the recruitment of more male participants due to cultural or religious norms or stereotypes. Likewise, another limitation is that the children's perceptions of the health coordinators are not included. Yet, the findings add valuable insights to developing PA programmes for families living in hard-to-reach or disadvantaged communities since recruitment in these areas is challenging [28]. To overcome recruitment-related challenges, researchers engaged in the programme for a prolonged period, and observations were conducted [23]. Another strength of this study is the use of multiple methods for data collection, and several researchers were involved in data collection, peer debriefing, triangulation, and reflexivity [37] to enhance trustworthiness [38].

## 7 Conclusion

Concerns about neighbourhood safety, social disorders, violence, and gang-related activities represent a significant barrier to accessing outdoor PA in disadvantaged communities. Utilising health coordinators who are informed about these concerns can represent a potential way forward to counteract inequalities in PA. This study suggests that families in disadvantaged areas have limited control over their PA participation due to feelings of unsafety and potential exclusion from mainstream society. Policies and PA programmes or practices that do not consider the pivotal role that health coordinators can play in these communities risk compromising the acceptability and sustainability of their endeavours. Further research is needed to explore which type of strategies or training health coordinators could receive to support communities that experience stigmatisation, trauma or social exclusion, as well as research that explores the potential social impact and sustainability aspects of including local leaders as health coordinators in their communities (e.g. adolescents that are well-recognized in their communities because their participation in sports or music).

## Ethical approval

The study was approved by the Regional Ethical Review Board in Stockholm (2022-02643-02). All participants provided consent before the interviews.

## Availability of data and material

Data is not publicly available due to containing information that could compromise the privacy of research participants. The data that support the findings of this study are available from the corresponding author upon reasonable request.

## Authors’ contributions

The study was conceived and designed by LF, M-LH, GN, and SA. LF and SA were responsible for data collection and analysis and drafting of the manuscript. GN and M-LH made critical revisions to the manuscript for important intellectual content. The final version of the manuscript was read and approved for submission by all authors.

## Funding

This work was supported by The Swedish Postcode Foundation. 10.13039/501100004047Karolinska Institutet provided Open Access funding.

## Declaration of competing interest

The authors declare that there is no conflict of interest.
